# Comparison of Current Systemic Combination Therapies for Metastatic Hormone-Sensitive Prostate Cancer and Selection of Candidates for Optimal Treatment: A Systematic Review and Bayesian Network Meta-Analysis

**DOI:** 10.3389/fonc.2020.519388

**Published:** 2020-09-18

**Authors:** Junru Chen, Yuchao Ni, Guangxi Sun, Banghua Liao, Xingming Zhang, Jinge Zhao, Sha Zhu, Zhipeng Wang, Pengfei Shen, Hao Zeng

**Affiliations:** Department of Urology, Institute of Urology, West China Hospital, Sichuan University, Chengdu, China

**Keywords:** metastatic prostate cancer, combination therapy, network meta-analysis, chemotherapy, androgen-deprivation therapy

## Abstract

**Objective:** To compare the efficacy and safety of current systemic combination therapies for patients with mHSPC and help select candidates for optimal treatment.

**Methods:** Databases of MEDLINE and EMBASE, Cochrane Central Register of Controlled Trials, and Clinical Trial.gov were searched for eligible studies. Direct and network meta-analysis were conducted to compare various systemic combination therapies and the surface under the cumulative ranking curve (SUCRA) was generated for treatment ranking. Subgroup analyses were performed according to the extent of metastasis. Adverse events (AEs) were compared among the effective treatments.

**Results:** Ten trials with 16 publications were included in this network meta-analysis. Direct and network meta-analysis consistently suggested that androgen-deprivation therapy (ADT) combined with docetaxel, abiraterone, enzalutamide, or apalutamide could significantly improve overall survival (OS) and failure-free survival (FFS) compared to ADT alone in men with mHSPC. SUCRA analysis demonstrated the superiority of ADT plus abiraterone or enzalutamide over other therapies. Subgroup analyses indicated that additional abiraterone to ADT had the highest ranking in patients with high-volume diseases or visceral metastases and enzalutamide plus ADT outperformed other treatments in patients with low-volume diseases or without visceral metastases. Different combination therapies had variable AE profiles and ADT in addition with docetaxel or abiraterone had the highest risk of AEs.

**Conclusion:** ADT plus docetaxel, abiraterone, enzalutamide, or apalutamide were associated with significantly improved survival in patients with mHSPC. ADT plus abiraterone or enzalutamide appeared to be the most effective treatments. Clinicians should balance the efficacy, potential AEs, and disease status to select the optimal treatment.

## Introduction

Prostate cancer (PCa) remains the most frequent solid malignancy among men in the United States, accounting for 20% of the new cancer cases in 2019 (1). Approximately of 5% patients diagnosed as PCa have distant metastases with a 5-year relative survival rate of only 30% (1). Over the past few decades, the standard of care for metastatic hormone-sensitive prostate cancer (mHSPC) has been androgen-deprivation therapy (ADT) through either surgical or medical castration. Unfortunately, almost all of the patients treated with ADT, or even ADT plus first-generation antiandrogen, ultimately become castration-resistant in a short time (2). It has been reported that no improvement in survival for men with mHSPC was observed for the last 20 years (3). Several randomized controlled trials (RCTs) have investigated the effectiveness of combination therapy in patients with mHSPC and suggested improved survival with additional bisphosphonates, celecoxib, docetaxel, or abiraterone alone or in combination (4–15). Therefore, the question arises spontaneously which is the optimal systemic treatment for men with mHSPC. Previous studies conducting comprehensive analyses demonstrated that abiraterone plus ADT might outperform other regimens in terms of survival (16, 17). However, recently, RCTs exploring the efficacy of ADT in addition to novel androgen receptor (AR) axis inhibitors such as enzalutamide and apalutamide, have emerged (18–20). To date, data regarding head-to-head comparisons of these combination therapies are limited. Thus, we conducted this updated systematic review and network meta-analysis aiming to compare the efficacy and safety of current combination systemic therapies for patients with mHSPC and help select candidates for optimal treatment.

## Methods

### Protocol and Registration

We developed a protocol defining search strategy and data analysis for this review, in accordance with the Preferred Reporting Items for Systematic Reviews and Meta-analyses (PRISMA) statement (21). The full protocol of this review was registered on PROSPERO (ID: CRD42019132967).

### Search Strategy

Databases of MEDLINE (1966–2019.9) and EMBASE (1974–2019.9) were searched through OVIDSP. Further searches were conducted through Cochrane Central Register of Controlled Trials (CENTRAL) (1948–2019.9) and Clinical Trial.gov (1999–2019.9). In addition, references of the included trials were screened for extra eligible trials. There was no restriction on language. The full search strategy was available in protocol and the last search was conducted on Sep 25, 2019.

### Eligibility and Exclusion Criteria

The eligibility criteria were (1) RCTs comparing systemic treatments for patients with mHSPC; (2) trials reporting survival outcomes. Exclusion criteria consisted of (1) patients with castration-resistant prostate cancer (CRPC); (2) patients without metastatic diseases; (3) cohorts, reviews, or case reports.

### Outcomes

The primary outcome was overall survival (OS), defined as the time from randomization to death from any cause. The secondary outcomes were failure-free survival (FFS) and adverse events (AEs). FFS was defined as the time from randomization until biological, radiographic/clinical progression, or death. For AEs, only AEs ≥ grade 3 were recorded to compare safety.

### Study Selection and Data Extraction

Two reviewers (JRC and YCN) independently examined the titles and abstracts of the publications meeting the eligibility criteria. For potentially eligible studies, full texts were assessed to identify the final included trials. Any discrepancies were reconciled by discussion or a third review (GXS). For included studies, we extracted information as follows: recruitment period, study population, follow-up time, number of patients, treatment comparisons, the definition of outcomes, performance status, patients age, prostate-specific antigen (PSA) value, T and N category, location of metastases, total Gleason score, disease volume, and type and incidence of AEs ≥ grade 3. We also collected the hazard ratio (HR) and corresponding 95% confidence interval (95% CI) for survival outcomes. For trials regarding enzalutamide and apalutamide, data in patients without prior docetaxel was used for analysis when it was available.

### Risk of Bias Assessment

Two reviewers evaluated the risk of bias of the included studies using RevMan software (version 5.3) based on Cochrane Handbook. The following items were assessed: (1) random sequence generation; (2) allocation concealment; (3) blinding of participants and personnel; (4) blinding of outcome assessment; (5) incomplete outcome data; (6) selective reporting; and (7) other sources of bias.

### Statistical Analysis

Direct comparisons were performed using RevMan 5.3. Fixed-effect model or random-effect model was used according to heterogeneity. Heterogeneity among trials in each comparison was evaluated with *I*^2^ statistic and χ^2^ test and *I*^2^ > 50% or χ^2^ *p* < 0.1 was considered as significant heterogeneity. The Bayesian network meta-analysis was conducted using Gemtc package within R program. Effect size for the Bayesian network meta-analysis was presented with 95% credible interval (CrI), as CrI was a more appropriate index than CI in Bayesian mixed treatment comparisons. Treatment rank probability was calculated and the surface under the cumulative ranking curve (SUCRA) was generated to describe the re-scaled mean ranking. SUCRA would be 1 if a treatment is certainly to be the best, whereas a treatment always ranks last would have a SUCRA value of 0 (22). Subgroup analyses for the most effective treatments were conducted according to disease volume and absence of visceral metastasis. High-volume disease was defined as the presence of visceral metastases or ≥4 bone lesions with ≥1 beyond the vertebral bodies and pelvis based on CHAARTED criteria (14). For trials with multiple reports, only the latest outcome data were used for analysis.

## Results

### Characteristics of Included Trials and Patients

**Figure 1:**
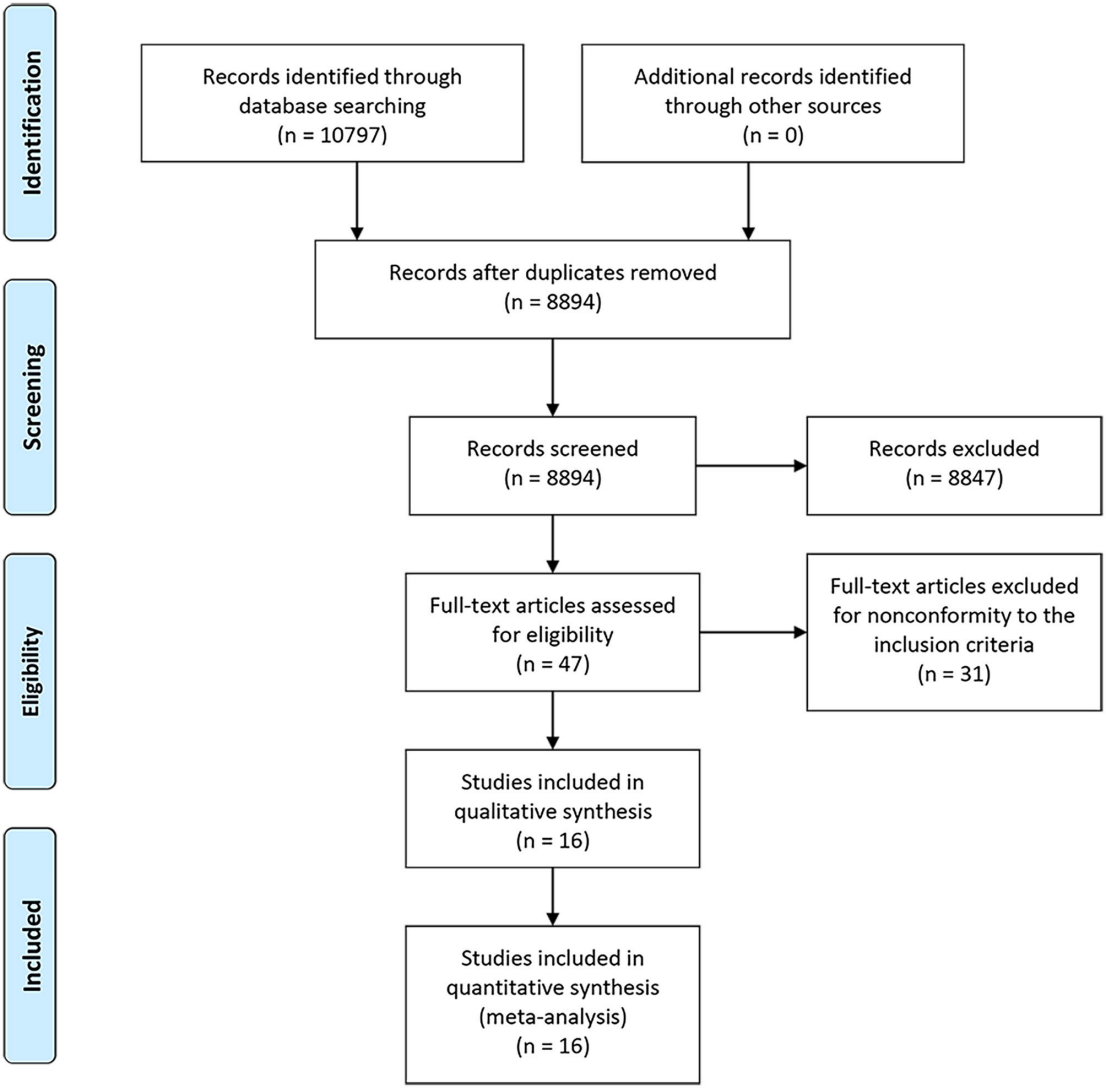
PRISMA flowchart of study selection.

A PRISMA flowchart summarizing the selection process of the eligible studies was shown in Figure 1. In total, 10 RCTs with 16 full-text articles and 11174 patients were included in the final quantitative meta-analysis (4–15, 18–20, 23). Characteristics of the included studies were provided in Table 1. Among the included trials, STAMPEDE used a multi-arm, multi-stage platform design to test efficacy of various combination treatments and shared one control arm. Five trials allowed maximal androgen blockade (7, 8, 11, 14, 18, 20). The median follow-up ranged from 14.4 to 138 months. All patients included were diagnosed as mHSPC, while men in LATITUDE trial were considered with high-risk disease which was defined as with at least two of the three following factors: Gleason score ≥ 8, bone lesions ≥ 3, and visceral metastasis (6). A majority of the patients had a favorable performance status and high Gleason score (≥8). Detailed baseline characteristics of the included patients were summarized in Table 2. The assessment of the risk of bias for included studies was shown in Supplementary Figure 1.

**Table 1:** Characteristics of the included studies.

**Trial**	**Study design**	**Recruitment period**	**Population**	**Median follow-up (month)**	**Treatment**	**Sample size**	**Definition of FFS**
STAMPEDE-Doc	RCT	October 2005–March 2013	mHSPC	43	ADT + Doc (75 mg/m^2^ every 3 weeks for 6 cycles) + prednisolone (10 mg daily)	362^#^	Time from randomization to biochemical failure, local/distant metastases, or death from PCa
					ADT	724^#^	
STAMPEDE-Abi	RCT	November 2011–January 2014	mHSPC	40	ADT + Abi (1000mg daily) + prednisolone (5 mg daily)	500^#^	Time from randomization to radiologic, clinical, biochemical progression, or death from prostate cancer
					ADT	502^#^	
GETUG-AFU15	RCT	October 2004–December 2008	mHSPC	84	ADT^*^+ Doc (75mg/m2 every 3 weeks up to 9 cycles) + corticosteroid premedication before, on the day, and day after infusion	192	Time from randomization to biochemical progression or radiographic progression or death.
					ADT^*^	193	
CHAARTED	RCT	July 2006–December 2012	mHSPC	28.9	ADT^*^+ Doc (75 mg/m^2^ every 3 weeks for 6 cycles) + dexamethasone (8 mg at 12, 3, and 1 h before docetaxel infusion)	397	Time from randomization to increasing symptoms of bone metastases, progression according to RECIST, or clinical deterioration due to cancer
					ADT^*^	393	
LATITUDE	RCT	February 2013–December 2014	High-risk mHSPC	30.4	ADT + Abi (1,000 mg daily) + prednisone (5 mg daily)	597	Time from randomization to radiographic progression or death from any cause
					ADT	602	
ENZAMET	RCT	March 2014–2017	mHSPC	34	ADT^*^+ Enza (160 mg daily)	563	Time from randomization to biochemical progression, clinical progression, death from any cause, or the last follow-up
					ADT^*^	562	
TITAN	RCT	December 2015–July 2017	mHSPC	22.7	ADT + Apa (240 mg daily)	525	Time from randomization to radiographic progression or death
					ADT	527	
ARCHES	RCT	March 2016–January 2018	mHSPC	14.4	ADT^*^+ Enza (160 mg daily)	574	Time from randomization to radiographic progression, assessed centrally or death from any cause
					ADT^*^	576	
ZAPCA	RCT	May 2008–December 2010	mHSPC	41.5	ADT^*^+ Bis (ZA 4 mg every 4 weeks for up to 2 years)	109	Time from randomization to biochemical progression, clinical progression, first SRE, death for any reason
					ADT^*^	110	
STAMPEDE-Bis	RCT	October 2005–March 2013	mHSPC	43	ADT + Bis (ZA 4 mg every 3 weeks for 6cycles, then every 4 weeks until 2 years)	366^#^	Time from randomization to biochemical failure, local/distant metastases or death from PCa
					ADT	724^#^	
STAMPEDE-Doc+Bis	RCT	October 2005–March 2013	mHSPC	43	ADT + Bis (ZA 4 mg every 3 weeks for 6 cycles, then every 4 weeks until 2 years) + Doc (75 mg/m^2^ every 3 weeks for 6 cycles) + prednisolone (10 mg daily)	365^#^	Time from randomization to biochemical failure, local/distant metastases, or death from PCa
					ADT	724^#^	
STAMPEDE-Cel	RCT	October 2005–April 2011	mHSPC	69	ADT + Cel (400 mg twice a day for 1 year)	188^#^	Time from randomization to biochemical failure, local/distant metastatic progression, or death from PCa
					ADT	377^#^	
STAMPEDE-Cel+Bis	RCT	October 2005–April 2011	mHSPC	69	ADT + Cel (400 mg twice a day for 1 year) + Bis (ZA 4 mg once every 3 weeks for 6 cycles then once every 4 weeks for 2 years)	190^#^	Time from randomization to biochemical failure, local/distant metastatic progression, or death from PCa
					ADT	377^#^	
CALGB	RCT	January 2004–May 2012	mHSPC	NA	ADT + Bis (ZA 4 mg every 4 weeks)	323	Time from randomization to bone progression, biochemical progression, or death
					ADT	322	
MRC PR05	RCT	June 1994–July 1998	mHSPC	138	ADT + Bis (sodium clodronate 2080 mg daily for up to 3 years)	155	NA
					ADT	156	
STAMPEDE-Abi/Doc	RCT	November 2011–March 2013	mHSPC	48	ADT + Abi (1,000 mg daily) + prednisolone (10 mg daily)	227^#^	Time from randomization to biochemical failure, local/distant metastatic progression, or death from PCa
					ADT + Doc (75 mg/m^2^ every 3 week for 6 cycles) + prednisolone (10 mg daily)	115^#^	

*RCT, randomized controlled trial; mHSPC, metastatic hormone-sensitive prostate cancer; Doc, docetaxel; Abi, abiraterone; Enza, enzalutamide; Apa, apalutamide; Bis, bisphosphonate; Cel, celecoxib; ZA, zoledronic acid; ADT, androgen-deprivation therapy; PCa, prostate cancer. NA, not applicable*.

* *Maximal androgen blockage allowed*.

# *Data of metastatic group were used*.

**Table 2:** Baseline characteristics of the included patients.

**Characteristic**	STAMPEDE-Doc^*^		STAMPEDE-Abi^*^		**GETUG-AFU15**		**CHAARTED**		**LATITUDE**		**ENZAMET**		**TITAN**		**ARCHES**		**ZAPCA**		STAMPEDE-Bis^*^		STAMPEDE-Doc+Bis^*^		STAMPEDE-Cel^*^		STAMPEDE-Cel+Bis^*^		**CALGB**		**MRC PR05**		STAMPEDE-Abi/Doc^*^	
	**ADT**	**CT**	**ADT**	**CT**	**ADT**	**CT**	**ADT**	**CT**	**ADT**	**CT**	**ADT**	**CT**	**ADT**	**CT**	**ADT**	**CT**	**ADT**	**CT**	**ADT**	**CT**	**ADT**	**CT**	**ADT**	**CT**	**ADT**	**CT**	**ADT**	**CT**	**ADT**	**CT**	**Doc**	**Abi**
Median age (years)	65	65	67	67	64	63	63	64	67	68	69	69.2	68	69	70	70	71.5	73	65	66	65	66	65	66	65	65	66.7	66.1	71	71	66	66
Median PSA (ng/ml)	67	70	56	51	25.8	26.7	52.1	50.9	NA	NA	NA	NA	4.02	5.97	5.1	5.4	375.2	328.0	67	59	67	63	67	57	67	70	6.8	6.9	NA	NA	58	55
ECOG=0	NA	NA	NA	NA	96%	99%	69.2%	69.8%	NA	NA	72.1%	71.9%	66%	62.5%	76.9%	78%	70%	67.9%	NA	NA	NA	NA	NA	NA	NA	NA	NA	NA	NA	NA	NA	NA
ECOG≥1	NA	NA	NA	NA	4%	1%	30.8%	30.2%	NA	NA	27.9%	28.1%	34%	37.5%	23.1%	21.8%	30%	32.1%	NA	NA	NA	NA	NA	NA	NA	NA	NA	NA	NA	NA	NA	NA
T stage ≤ T2	13%	10%	10%	9%	NA	NA	NA	NA	NA	NA	29.1%	26.3%	26%	35.8%	NA	NA	NA	NA	13%	11%	13%	12%	12%	8%	12%	13%	NA	NA	NA	NA	13%	10%
T stage≥T3	82%	84%	85%	86%	NA	NA	NA	NA	NA	NA	45.9%	50.6%	62.6%	54.5%	NA	NA	NA	NA	82%	83%	82%	80%	81%	84%	81%	81%	NA	NA	NA	NA	85%	84%
N stage=N0	44%	44%	46%	45%	NA	NA	NA	NA	NA	NA	42.2%	40.1%	41%	40.4%	NA	NA	NA	NA	44%	44%	44%	45%	45%	46%	45%	45%	NA	NA	NA	NA	44%	42%
N stage=N1	50%	50%	50%	50%	NA	NA	NA	NA	NA	NA	34.5%	36.4%	34.9%	37.9%	NA	NA	NA	NA	50%	51%	50%	49%	49%	47%	49%	48%	NA	NA	NA	NA	52%	54%
Visceral metastases	4%	5%	3%	3%	12%	15%	16.8%	14.4%	19%	20%	12%	11%	14.6%	11.3%	49.6%	46.7%	NA	NA	4%	5%	4%	4%	4%	3%	4%	6%	6%	7%	NA	NA	NA	NA
GS <8	24%	19%	23%	23%	41%	45%	26.4%	29.5%	2.2%	2.7%	29%	27%	32.1%	33.1%	32.5%	29.8%	16.4%	19.3%	24%	21%	24%	20%	25%	26%	25%	27%	36%	39%	NA	NA	19%	25%
GS≥8	68%	74%	75%	74%	59%	55%	61.8%	60.7%	97%	98%	57%	60%	67.9%	66.9%	64.8%	67.2%	83.6%	80.7%	68%	71%	68%	72%	66%	65%	66%	62%	58%	58%	NA	NA	81%	75%
Low volume	NA	NA	NA	NA	53%	52%	36.4%	33.8%	NA	NA	47%	48%	36.4%	38.1%	35.2%	38.3%	NA	NA	NA	NA	NA	NA	NA	NA	NA	NA	NA	NA	NA	NA	NA	NA
High volume	NA	NA	NA	NA	47%	48%	63.6%	66.2%	NA	NA	53%	52%	63.6%	61.9%	64.8%	61.7%	NA	NA	NA	NA	NA	NA	NA	NA	NA	NA	NA	NA	NA	NA	NA	NA

*Doc, docetaxel; Abi, abiraterone; Bis, bisphosphonate; Cel, celecoxib; ADT, androgen-deprivation therapy; CT, combination therapy; PSA, prostate-specific antigen; ECOG, Eastern Cooperative Oncology Group; GS, Gleason score*.

* *Data of overall population were used*.

### Direct Comparisons

**Figure 2:**
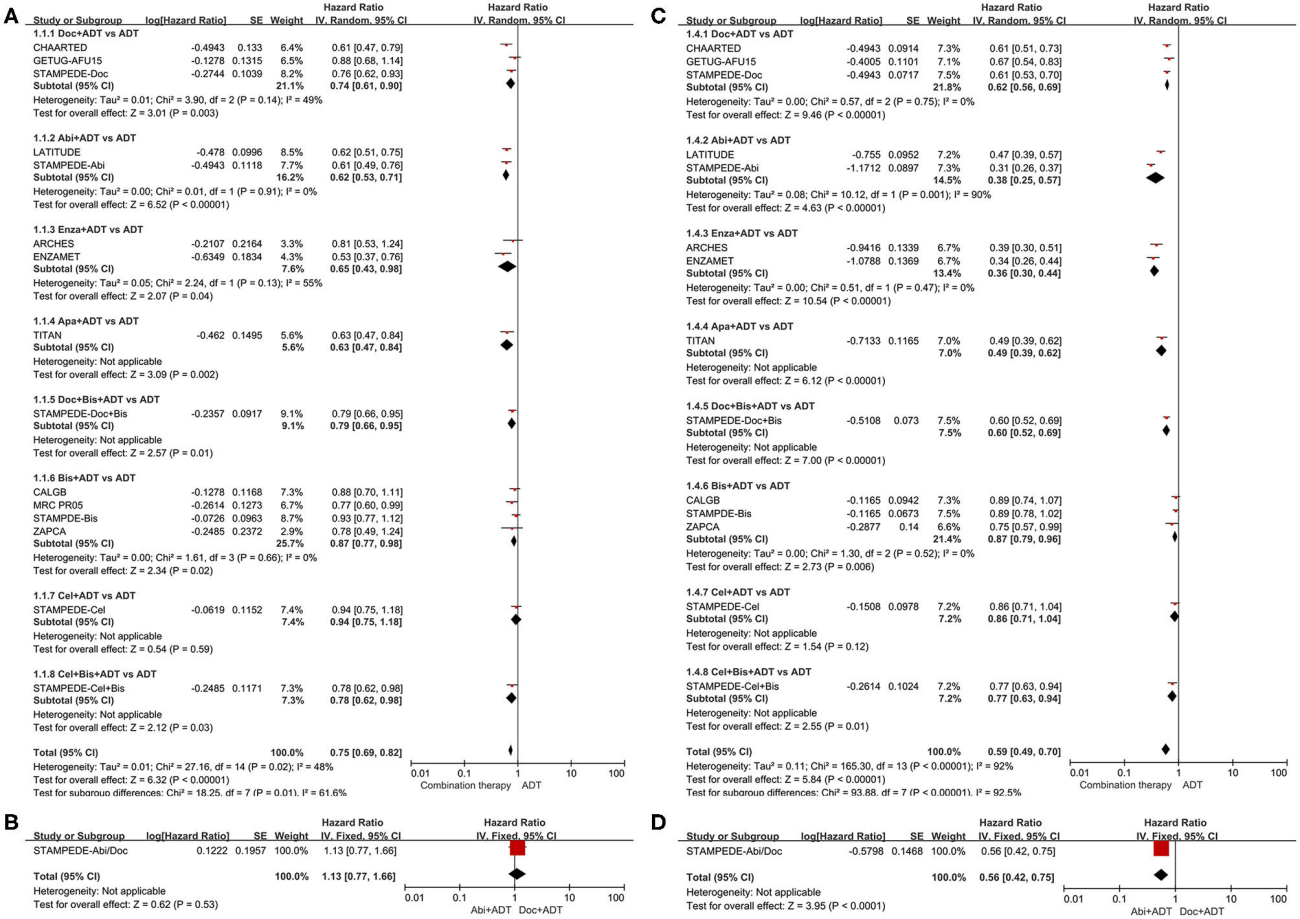
Forest plots of direct comparisons. **(A)** Effect of combination therapies compared to ADT alone on overall survival. **(B)** Effect of ADT plus abiraterone compared to ADT plus docetaxel on overall survival. **(C)** Effect of combination therapies compared to ADT alone on failure-free survival. **(D)** Effect of ADT plus abiraterone compared to ADT plus docetaxel on failure-free survival. Doc, docetaxel; Abi, abiraterone; Enza, enzalutamide; Apa, apalutamide; Bis, bisphosphonate; Cel, celecoxib; ADT, androgen-deprivation therapy.

As shown in Figures 2A,B, ADT in addition to docetaxel (HR = 0.74, 95%CI = 0.61–0.90), abiraterone (HR = 0.62, 95%CI = 0.53–0.71), enzalutamide (HR = 0.65, 95%CI = 0.43–0.98), apalutamide (HR = 0.63, 95%CI = 0.47–0.84), docetaxel + bisphosphonate (HR = 0.79, 95%CI = 0.66–0.95), bisphosphonate (HR = 0.78, 95%CI = 0.77–0.98), or bisphosphonate + celecoxib (HR = 0.78, 95%CI = 0.62–0.98) showed benefit in OS compared to ADT alone in patients with mHSPC. However, celecoxib plus ADT could not improve OS compared to ADT alone (HR = 0.94, 95%CI = 0.75–1.18). Moreover, abiraterone plus ADT showed no superiority than docetaxel plus ADT in men with mHSPC with a HR of 1.13 (95%CI = 0.77–1.66).

In terms of FFS (Figures 2C,D), additional docetaxel (HR = 0.62, 95%CI = 0.56–0.69), abiraterone (HR = 0.38, 95%CI = 0.25–0.57), enzalutamide (HR = 0.36, 95%CI = 0.30–0.44), apalutamide (HR = 0.49, 95%CI = 0.39–0.62), or docetaxel + bisphosphonate (HR = 0.60, 95%CI = 0.52–0.69) showed strong benefits compared to ADT alone in men with mHSPC. Besides, additional bisphosphonate (HR = 0.87, 95%CI = 0.79–0.96) or bisphosphonate + celecoxib (HR = 0.77, 95%CI = 0.63–0.94) showed moderate benefits compared to ADT alone. No statistically significant improvement in FFS was observed with additional celecoxib (HR = 0.86 95%CI = 0.79–1.04). Obvious net benefit was seen with abiraterone plus ADT (HR = 0.56, 95%CI = 0.42–0.75) compared to docetaxel plus ADT.

### Network Comparisons

**Figure 3:**
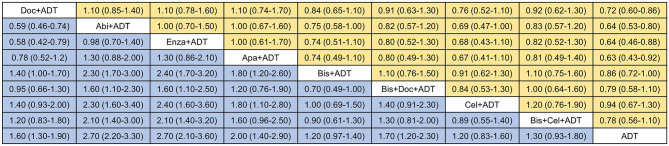
Network comparisons of effect of systemic therapies on overall survival (yellow) and failure-free survival (blue). Results are the HRs with 95% CrIs from the network meta-analysis between the row intervention and column intervention. Doc, docetaxel; Abi, abiraterone; Enza, enzalutamide; Apa, apalutamide; Bis, bisphosphonate; Cel, celecoxib; ADT, androgen-deprivation therapy.

**Figure 4:**
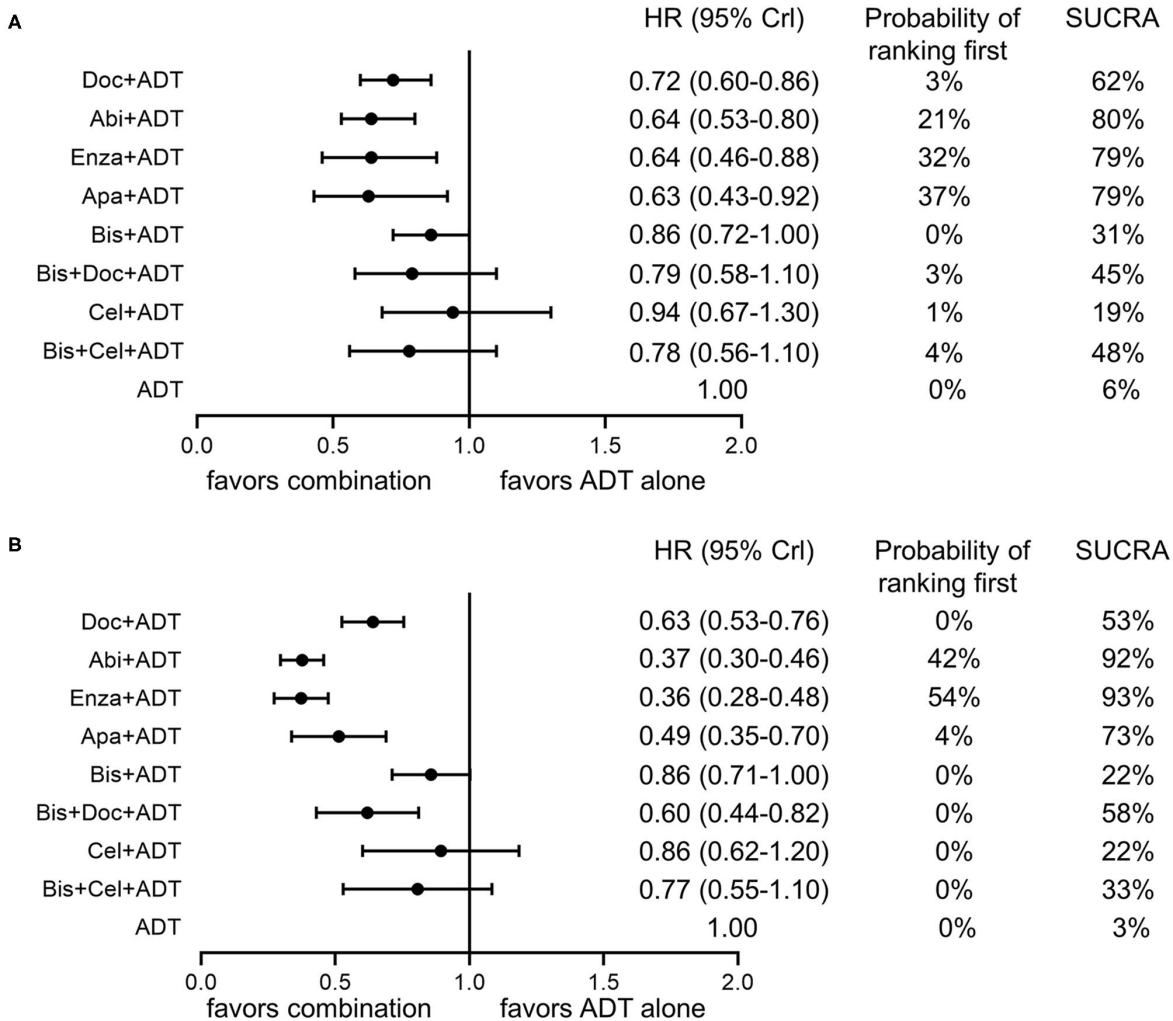
Forest plots of network comparisons of effect of combination therapies compared to ADT alone on overall survival **(A)** and failure-free survival **(B)**. Rankings are presented as probabilities of ranking first and SUCRA. Doc, docetaxel; Abi, abiraterone; Enza, enzalutamide; Apa, apalutamide; ADT, androgen-deprivation therapy.

As shown in Figures 3, 4A, ADT in addition to docetaxel (HR = 0.72, 95%CrI = 0.60–0.86), abiraterone (HR = 0.64, 95%CrI = 0.53–0.80), enzalutamide (HR = 0.64, 95%CrI = 0.46–0.88) or apalutamide (HR = 0.63, 95%CrI = 0.43–0.92) showed significant improvement in OS compared to ADT alone. While, according to the results of network meta-analysis, ADT with additional bisphosphonate (HR = 0.86, 95%CrI = 0.72–1.00), docetaxel + bisphosphonate (HR = 0.79, 95%CrI = 0.58–1.10), celecoxib (HR = 0.94, 95%CrI = 0.67–1.30), or bisphosphonate + celecoxib (HR = 0.78, 95%CrI = 0.56–1.10) showed no survival benefit compared to ADT alone. No one combination therapy showed significant superiority than another in terms of OS.

For FFS (Figures 3, 4B), obvious survival benefit was associated with ADT in addition to abiraterone (HR = 0.37, 95%CrI = 0.30–0.46), enzalutamide (HR = 0.36, 95%CrI = 0.28–0.48), or apalutamide (HR = 0.49, 95%CrI = 0.35–0.70). Moderate benefit was observed with combination of ADT plus docetaxel or docetaxel + bisphosphonate (HR = 0.60, 95%CrI = 0.44–0.82). No significant benefit was seen with ADT in addition to bisphosphonate (HR = 0.86, 95%CrI = 0.71–1.00), celecoxib (HR = 0.86, 95%CrI = 0.62–1.20) or bisphosphonate + celecoxib (HR = 0.77, 95%CrI = 0.55–1.10) compared to ADT alone. ADT with additional abiraterone or enzalutamide showed superiority over other combination therapies except for ADT plus apalutamide.

### Treatment Rankings

As shown in Figure 4A, ADT plus abiraterone, enzalutamide or apalutamide seemed to be the three most effective treatments with a possibility of 21, 32, 37% ranking first, respectively, and a SUCRA of 80, 79, and 79%, respectively, in terms of OS. As for FFS (Figure 4B), ADT plus abiraterone or enzalutamide seemed to be the most effective treatments with a probability of 42 and 54% ranking first, respectively, and a SUCRA of 92 and 93%, respectively.

### Subgroup Analysis for the Four Effective Therapies

**Figure 5:**
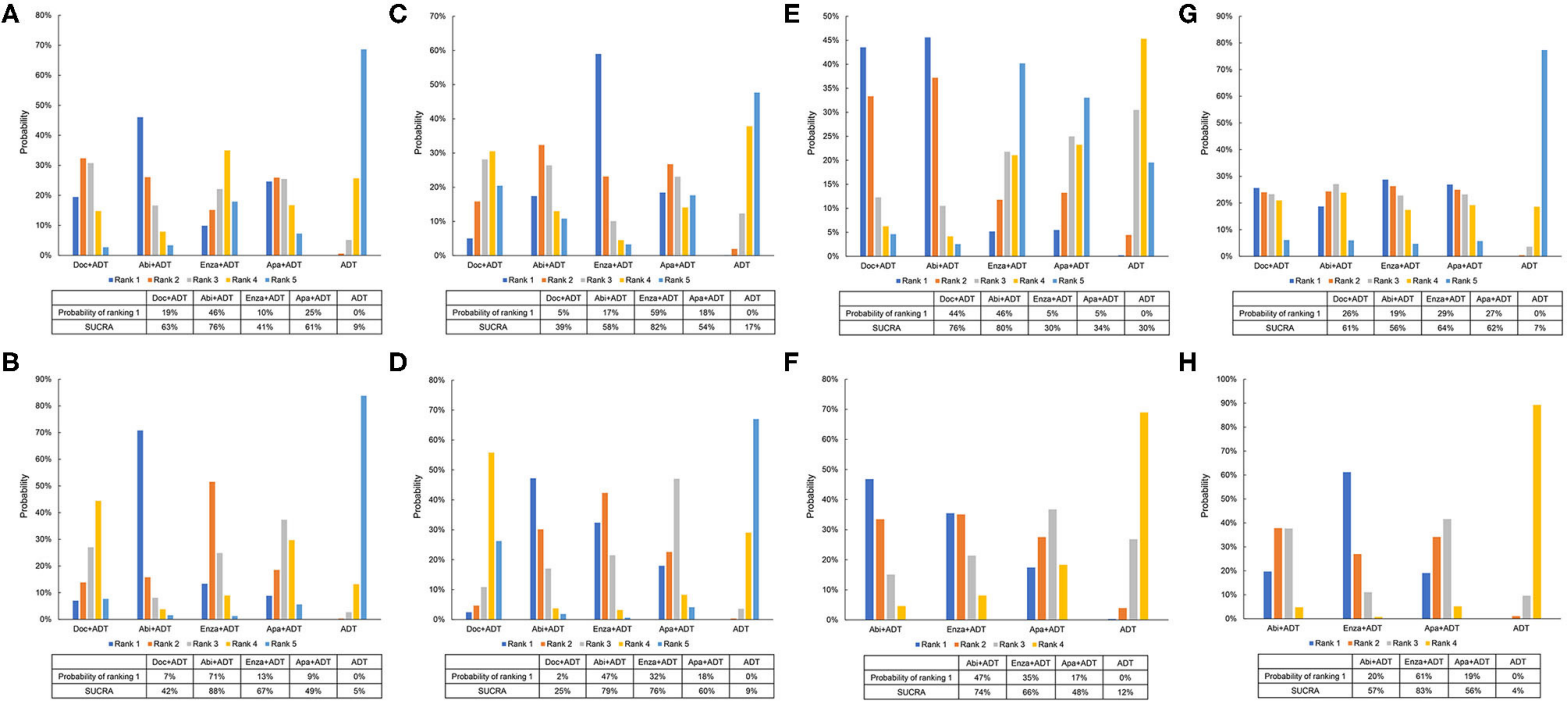
Ranking probabilities of effective therapies for overall survival and failure-free survival. **(A)** Ranking probabilities of effective therapies for overall survival in patients with high-volume diseases. **(B)** Ranking probabilities of effective therapies for failure-free survival in patients with high-volume diseases. **(C)** Ranking probabilities of effective therapies for overall survival in patients with low-volume diseases. **(D)** Ranking probabilities of effective therapies for failure-free survival in patients with low-volume diseases. **(E)** Ranking probabilities of effective therapies for overall survival in patients with visceral metastases. **(F)** Ranking probabilities of effective therapies for failure-free survival in patients with visceral metastases. **(G)** Ranking probabilities of effective therapies for overall survival in patients without visceral metastases. **(H)** Ranking probabilities of effective therapies for failure-free survival in patients without visceral metastases. Doc, docetaxel; Abi, abiraterone; Enza, enzalutamide; Apa, apalutamide; ADT, androgen-deprivation therapy.

For patients with high-volume disease, abiraterone in addition with ADT seemed to be the most effective therapy with a probability of 46% ranking first and a SUCRA of 76% in terms of OS and 71 and 88%, respectively, in terms of FFS (Figures 5A,B). For those with low-volume disease, enzalutamide plus ADT showed superiority to other therapies with a probability of 59% ranking first and a SUCRA of 82% in terms of OS (Figure 5C). While, in terms of FFS, additional abiraterone to ADT outperformed others with a probability of 47% ranking first and a SUCRA of 79% (Figure 5D).

For men with visceral metastasis, abiraterone plus ADT seemed to be the best option with a probability of 46% ranking first and a SUCRA of 80% in terms of OS and 47% and 74%, respectively, in terms of FFS (Figures 5E,F). For men without visceral metastasis, enzalutamide plus ADT was superior to other regimens with a probability of 29% ranking first and a SUCRA of 64% in terms of OS and 61% and 83%, respectively, in terms of FFS (Figures 5G,H).

### Adverse Events

Among the four effective treatments, docetaxel plus ADT and abiraterone plus ADT had the highest risk of overall AEs ≥ grade 3 with the same SUCRA of 79%. Docetaxel in addition to ADT had the highest risk of febrile neutropenia and anemia with a SUCRA of 93 and 87%, respectively. Abiraterone plus ADT had the highest risk of hypertension, cardiac disorder, and alanine transaminase (ALT) increase with a SUCRA of 90, 93, and 83%, respectively. Apalutamide plus ADT was associated with the highest risk of fracture with a SUCRA of 65%. ADT in addition with docetaxel, abiraterone or enzalutamide had a similar risk of nervous system disorder (SUCRA: 65, 62, and 65%, respectively). On the contrary, apalutamide plus ADT had the lowest risk of overall AEs, anemia and hypertension (SUCRA: 28, 25, and 24%, respectively). Abiraterone plus ADT had the lowest risk of fracture with a SUCRA of 42%. Enzalutamide plus ADT had the lowest risk of febrile neutropenia and ALT increase (SUCRA: 33 and 29%, respectively). Docetaxel plus ADT had the lowest risk of cardiac disorder with a SUCRA of 26%.

## Discussion

Several previous meta-analyses have indirectly compared various systemic therapies in terms of survival benefit in mHSPC patients (16, 17, 24). Recently, ADT in combination with novel AR-targeted therapies including enzalutamide and apalutamide has been reported to improve survival in patients with mHSPC, which provided extra effective systemic treatment choices for this population. The present network meta-analysis comprehensively evaluated the efficacy of current systemic therapies for men with mHSPC based on the most up-to-date results from RCTs. We found that ADT in addition to docetaxel, abiraterone, enzalutamide or apalutamide could significantly prolong both FFS and OS compared to ADT alone in men with mHSPC and abiraterone and enzalutamide appeared to be the most effective treatments for this setting.

Our results were in consistence with previous studies which reported the superiority of docetaxel and abiraterone to standard ADT in patients with mHSPC (16, 17, 24, 25). However, whether abiraterone is better than docetaxel in this population remains a debate. Recently, the STAMPEDE trial directly compared ADT plus docetaxel and ADT plus abiraterone in men with mHSPC based on its multi-arm design and showed an advantage of ADT plus abiraterone on FFS but not OS compared to ADT plus docetaxel (15). This result should be interpreted cautiously, as the trial was not designed to directly compare these two therapies and might be underpowered to detect the differences. Our findings also indicated that patients with mHSPC could benefit more from ADT in combination with abiraterone with respect to FFS, but had similar OS with those received ADT plus docetaxel. Network meta-analysis is considered to be more powerful in detecting differences between treatments because of more patients included and the combination of direct and indirect comparisons. However, it should be noted that the differences in patient baseline characteristics might have an impact on the results of comparisons. The sample size of the GETUG-AFU15 trial was relatively small and approximately half of the patients had low-risk diseases according to Glass risk stratification or low-volume diseases based on CHAARTED criteria, which might attenuate the effects of docetaxel (8). While, as mentioned previously, patients in the LATITUDE trial all had high-risk diseases, which might inversely overestimate the benefit of abiraterone (6). Therefore, SUCRA analysis demonstrated that abiraterone had a higher probability to be the preferred treatment than docetaxel despite the lack of statistical significance in network comparison. *Post-hoc* analyses of the current RCTs based on consistent stratification criteria and results of direct comparisons from clinical trials are needed to provide more reliable evidence.

Enzalutamide and apalutamide are novel potent AR-targeted agents and have been proved their efficacy in mHSPC setting by recent RCTs (18–20). To the best of our knowledge, this is the first network meta-analysis comparing enzalutamide and apalutamide with other systemic combination regimens in men harboring mHSPC. The results suggested that both enzalutamide and apalutamide could significantly improve FFS as well as OS compared to ADT alone. Moreover, enzalutamide and apalutamide had similar efficacy as abiraterone over other regimens based on SUCRA analysis. The ENZAMET trial allowed early use of docetaxel and showed significant improvement in survival in overall population and patients without early docetaxel but limited effects on survival in patients with early docetaxel (20). In our network meta-analysis, given the established role of docetaxel in the management of mHSPC, we extracted data from subgroup without early docetaxel to preclude the influence of docetaxel and better compare the effects of enzalutamide with other therapies. The benefit of ADT in addition with enzalutamide was also confirmed in the ARCHES trial (18). However, due to relatively short follow-up time, no statistically significant improvement in OS was observed in the ARCHES trial.

The extent of disease has a strong association with the prognosis of patients and could result in different responses to therapies (14). Therefore, selection of candidates based on disease extent to receive proper treatment is essential to reach maximal benefits. Despite similar efficacy of abiraterone and enzalutamide for overall mHSPC setting, the subgroup analyses in the present study suggested the superiority of abiraterone in addition with ADT in patients with high-volume disease or visceral metastasis and enzalutamide plus ADT in patients with low-volume disease or without visceral metastasis. Compared to other trials, the two trials (ENZAMET and ARCHES) on enzalutamide seemed to be complicated with more confounding factors such as prior and concomitant use of docetaxel, various standard nonsteroidal antiandrogen drugs and a high proportion of metachronous metastatic PCa (18, 20). Previous studies have demonstrated that different standard nonsteroidal antiandrogens had varied impacts on prognosis and metachronous metastatic PCa had poorer response to chemohormonal therapy and shorter survival than *de novo* metastatic PCa, which might partially explain the differences in survival benefit of enzalutamide on patients with different extensive diseases (26–28).

Making the optimal treatment decision is not only about pursuing maximal survival benefit but also decreasing AEs. According to our analyses, additional docetaxel and abiraterone were associated with a higher risk of overall AEs. Additional docetaxel was associated with a higher risk of myelosuppression, abiraterone had a higher risk of hypertension, cardiac disorder and ALT increase, and apalutamide had a higher risk of fracture. Patients in the LATITUDE trial had a higher incidence of AEs than those in other trials, which might result from poorer baseline characteristics (6). These results could help clinicians select therapies for patients under specific physical conditions to reduce and even avoid some treatment-induced AEs. Moreover, enzalutamide and apalutamide require no glucocorticoid which might be safer in patients with poorly controlled diabetes or heart failure.

This network meta-analysis is not without limitations. First, heterogeneities existed in the baseline characteristics of the include trials and patients. Maximal androgen blockage was allowed in five trials and prior or concomitant use of docetaxel in three trials. Besides, as shown in Table 1, several trials have different definitions of FFS with others and reported biochemical and radiographic progression separately. Also, the definition of high-volume disease in the TITAN trial was a little bit different from CHAARTED criteria. Third, fewer studies and smaller sample sizes in subgroup analysis could attenuate the power to detect differences in survival and increase bias. Fourth, the STAMPEDE trial used a multi-arm, multi-stage platform design, in which combination therapy groups were compared with the same control group. Moreover, due to limited comparisons and data on AEs, some of the results might differ from the real world. It is worth noting that the cardiotoxicity of docetaxel is not rare in spite of the lowest risk according to our results (29). Large prospective trials are needed to provide more potent evidence.

## Conclusion

ADT with additional docetaxel, abiraterone, enzalutamide, or apalutamide could significantly improve survival compared to ADT alone in patients with mHSPC. ADT plus abiraterone or enzalutamide appeared to be the most effective treatments for overall population. Abiraterone plus ADT showed superiority in patients with high-volume disease or visceral metastasis and enzalutamide plus ADT outperformed other treatments in patients with low-volume disease or without visceral metastasis. Additional docetaxel and abiraterone were associated with a higher risk of AEs. Clinicians should balance the efficacy, safety, and even the extent of disease to select the optimal treatment.

## Data Availability Statement

All datasets analyzed for this study are included in the article and the supplementary files.

## Author Contributions

JC and YN designed the study, analyzed the data, and wrote the manuscript. BL and GS helped literature searching and data analysis. SZ, XZ, and ZW helped prepare materials and revise the manuscript. PS and HZ supervised the project and revised the manuscript. All authors read and approved the final manuscript. All authors contributed to the article and approved the submitted version.

## Conflict of Interest

The authors declare that the research was conducted in the absence of any commercial or financial relationships that could be construed as a potential conflict of interest.

## Supplementary Material

Supplementary Figure 1 Assessment of risk of bias of the included studies.
