# Supplementary figures and images for: Comparison of Current Systemic Combination Therapies for Metastatic Hormone-Sensitive Prostate Cancer and Selection of Candidates for Optimal Treatment: A Systematic Review and Bayesian Network Meta-Analysis
Source: Front Oncol. 2020 Sep 18;10:519388. doi: 10.3389/fonc.2020.519388 (PMC7531177; doi:10.3389/fonc.2020.519388)

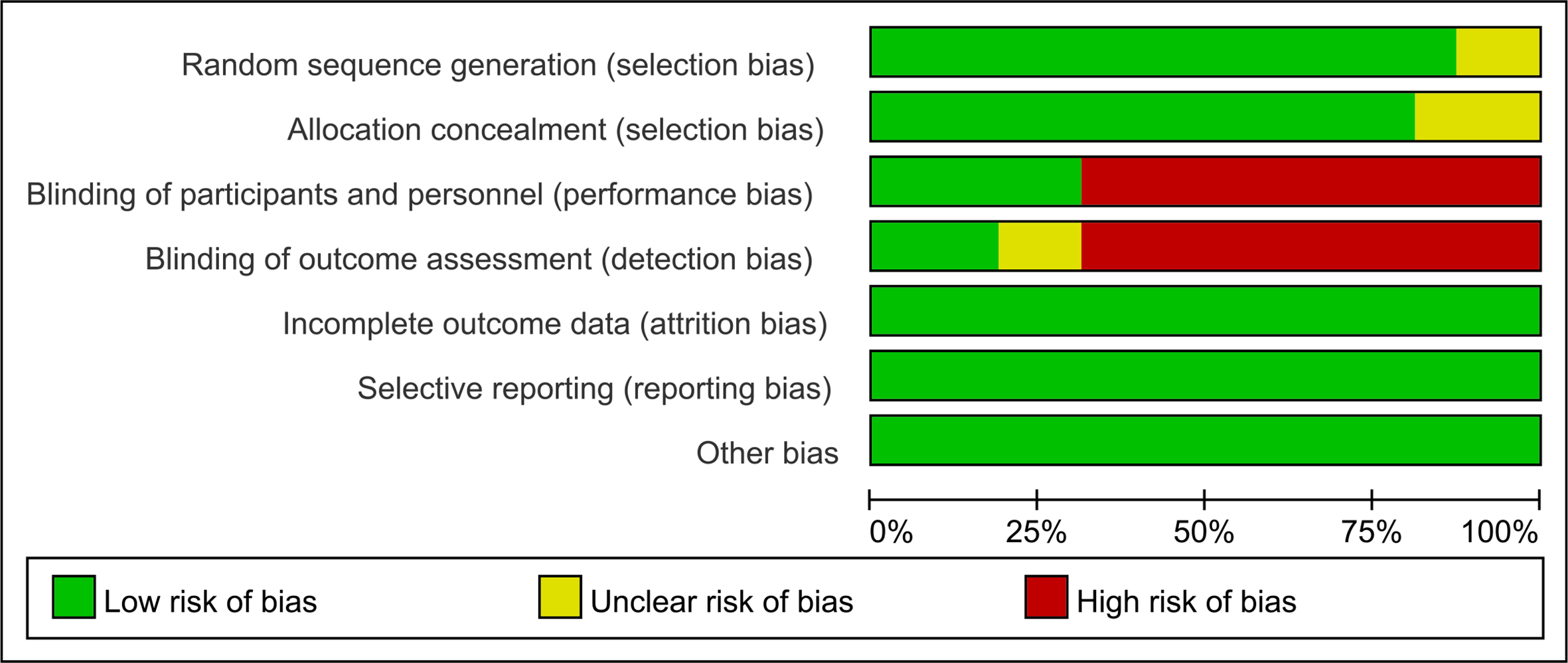

Supplement: Supplementary Figure 1 — Assessment of risk of bias of the included studies. [file Image_1.TIF]
